# Designing Flood Insurance to Maximize Public Benefits in High‐Risk Areas

**DOI:** 10.1111/risa.70305

**Published:** 2026-08-11

**Authors:** Michael Bourdeau‐Brien, Jason Thistlethwaite, Mathieu Boudreault, Gabriel Morin, Daniel Henstra, Jacob Chenette

**Affiliations:** ^1^ Département de finance, assurance et immobilier Université Laval Quebec City Québec Canada; ^2^ School of Environment, Enterprise and Development University of Waterloo Waterloo Ontario Canada; ^3^ Département de mathématiques Université du Québec à Montréal Montréal Québec Canada; ^4^ Department of Political Science University of Waterloo Waterloo Ontario Canada

**Keywords:** adverse selection, disaster financial assistance, flood insurance, high risk, policy objectives

## Abstract

Maximizing the public benefits of flood insurance in high‐risk areas requires premiums that are affordable and measures that minimize the concentrated demand that causes adverse selection. Governments make policy choices for flood insurance based on their national interests, and these choices lead to variation in how this balance is achieved. This article evaluates the influence of different design elements on the policy objectives identified for the Canadian Flood Insurance Program (CFIP), which include affordability, adequate compensation, and market participation. Combinations of deductibles, limits, exclusions, and subsidies were evaluated using a novel dataset of flood losses from 700,000 simulated flood events to determine how they shape the trade‐offs between these objectives. The optimal design includes a progressive subsidy, premium caps, a moderate deductible, a coverage limit, and exclusions for the highest risk households. These findings offer a useful comparison with international practices and insights for policymakers seeking to maximize the benefits of flood insurance in Canada.

## 1 Introduction

Damage from natural hazards is growing worldwide due to climate change and increasing economic exposure (Coronese et al. 2019). Catastrophe insurance is a risk management tool that complements loss prevention, reduction, and avoidance techniques. Insurance provides a financial safety net against large‐scale losses that could otherwise severely affect households and communities, reduces the financial burden for publicly funded disaster assistance, and creates a price signal that encourages individuals and communities to invest in risk mitigation (Thistlethwaite 2016). Flood insurance is arguably the most common type of catastrophe insurance, which is hardly surprising given that hydrological disasters such as floods are the most frequent and costly class of natural hazards worldwide (Shen and Hwang 2019).

Flood insurance design varies because government intervention or a public–private partnership is typically required to offset costs to private insurers that are exposed to significant financial volatility through adverse selection and concentrated loss. Paudel (2012) documents large disparities in the design and distribution of responsibility between governments, insurers, local governments, and property owners for the administration and capitalization of flood insurance. This diversity is a result of public policy choices that prioritize some flood insurance objectives over others (Hudson et al. 2019; Jaffee 2015; Kunreuther 1998; Mysiak and Pérez‐Blanco 2016). For example, models that use fully risk‐adjusted premiums prioritize market stability by making sure insurers are adequately compensated but can struggle to achieve market penetration in high‐risk areas because of cost‐prohibitive premiums (Botzen and van den Bergh 2008; Kunreuther and Michel‐Kerjan 2009). Other models adopt a principle of “solidarity” that seeks to share the cost of flooding across a political jurisdiction regardless of whether some in high‐risk areas will take up more resources than others (Fournier et al. 2018; Charpentier et al. 2022). These systems use flat or minimally risk‐adjusted premiums supported through government subsidization that encourages market participation in high‐risk areas (Atreya et al. 2015).

Market participation is important as it leads to a more diverse pool of insured households and helps insurers accurately predict claims by lowering the safety margins incorporated into premiums, which results in more competitive pricing. High participation in high‐risk areas also reduces governmental reliance on post‐event disaster relief (Palm 1998). A model of insurance that prioritizes availability and affordability through rate regulation can, however, lead to adverse selection whereby only those in high‐risk areas purchase coverage. Adverse selection creates financial instability for public or private catastrophe systems as there is a greater chance that damage from a hazard in a high‐risk zone exceeds the premiums collected (e.g., Bradt et al. 2021).

To balance market participation and manage adverse selection, insurance systems embrace design elements, such as deductibles, coverage limits, exclusions, subsidies, and premium caps. These elements help to offset concentrations of risk that disincentive participation in the market among policyholders, insurers, and governments. For example, deductibles (and limits) can be increased or decreased in high‐risk areas to ensure that the financial liability of losses concentrated in high‐risk areas is fairly distributed to property owners who use coverage more frequently. This encourages insurers to participate in the market by providing predictability to the cost of coverage in a location exposed to volatility. Market participation also increases as premiums are more affordable in low‐ and moderate risk zones because high‐risk households contribute more for their exposure by paying the deductible or self‐insuring if damage exceeds the limit (Paudel et al. 2015). Market exclusions limit the exposure of insurers to high‐risk areas as a means of mitigating adverse selection and lowering the price of insurance for low‐ and moderate‐risk areas (i.e., Priest 2003) but usually require government disaster assistance to address the coverage gap.

Although these measures diversify concentrations of risk, they can lower the adequacy of compensation households receive as losses go uninsured, especially in high‐risk areas (e.g., losses that exceed coverage limits). The perception among households that coverage is inadequate can reduce demand and market participation. Subsidies can be used to lower the cost of premiums, thereby improving perceptions about the utility of coverage with high deductibles or low coverage limits. Subsidies can comprise either a direct contribution or a cap on coverage where the difference between the subsidized and risk‐adjusted prices is made up with the subsidy. This strategy distributes risk between property owners and governments (who pay the subsidy) as a means of improving affordability and increasing market participation to diversify the risk pool. Ultimately, governments must balance the objectives of affordability, coverage adequacy, and market participation for the design of the insurance system based on a political consensus defined by key interests, including policymakers, industry, and property owners.

This article presents a novel investigation into the implications of different design choices for the Canadian Flood Insurance Program (CFIP), which is under development at the time of writing. This evaluation is based on an original dataset of property‐level flood losses arising from 700,000 simulated flood events. Flood loss estimates allow for the calculation of actuarially fair insurance premiums that can be adjusted using deductibles, limits, exclusions, and subsidies to achieve different policy objectives such as affordability, market participation, and adequate compensation. The next section of the article provides background on the emergence of flood insurance in Canada, including the public policy objectives considered to generate a public benefit. The third section provides details on the flood risk model and the development of a baseline insurance design scenario. Section 4 discusses the measurable criteria used to assess public policy objectives and how changes in insurance design impact these criteria. The fifth section discusses the implications of insurance design scenarios and calibrates design elements to maximize alignment with the public policy objectives. The sixth and last section provides a conclusion, identifies the study's contributions to existing literature, and areas for future research.

## 2 Flood Insurance in Canada

Canada has historically relied on publicly funded disaster financial assistance (DFA) to help households recover from natural hazard damage. Flood losses account for approximately 75% of DFA's historical costs, and these losses are expected to increase in the future (Office of the Parliamentary Budget Officer 2016). The use of the DFA program to fund flood recovery is widely viewed as unsustainable due to increasing costs and the moral hazard of funding rebuilding without incentives for risk reduction or relocation (Council of Canadian Academies 2022; Oulahen 2015).

In 2015, private insurers expanded their coverage to include damage from overland flooding in response to reputational and regulatory pressure from governments and consumers frustrated by the reliance on DFA programs (Thistlethwaite 2016). In the years since, the availability of insurance coverage for flood damage has expanded with approximately 90% of households now able to purchase some form of policy (Insurance Bureau of Canada 2023). In high‐risk areas, however, flood insurance is often unavailable, prohibitively expensive, or provides inadequate coverage (Darlington and Yiannakoulias 2022).

On the basis of the findings from a National Task Force on Flood Insurance and Relocation, the Government of Canada (Task Force) announced in March 2023 that it will establish a flood insurance program that offers affordable protection to homeowners in high‐risk areas (Government of Canada 2023). The 2024 federal budget allocated $15 million to the Canada Mortgage and Housing Corporation (CMHC) to advance the creation of a subsidiary that would deliver a flood reinsurance program and a separate insurance subsidy for households at high risk of flooding (Canada 2024).

The public value of the CFIP is dependent on achieving a set of objectives identified by the National Task Force that are difficult to balance in areas with a high concentration of risk. Specifically, the Task Force argued that insurance must be both affordable and adequate with compensation that ensures sufficient participation to diversify the risk pool. This balance could be achieved through a series of design elements, including deductibles, limits, exclusions, and subsidies, that prompt a redistribution of the financial burden of losses among governments, insurers, and households. Costs of the CFIP are offset by higher deductibles, coverage limits, and market exclusions in high‐risk areas. In turn, government subsidies are used to ensure premiums for households in high‐risk areas are affordable. Similar studies have documented how flood insurance systems in the United Kingdom, Germany, and the United States increase or decrease availability and affordability based on these design elements (Hudson et al. 2019; Kousky 2018; Kunreuther 1996; Surminksi et al. 2020). This study is the first of its kind in Canada and offers insights for policymakers seeking to maximize the CFIP's public benefits.

## 3 Data and Methods

### 3.1 Flood Risk Model

The first step in evaluating insurance design involves assessment of flood losses among the high‐risk properties that will be targeted by the CFIP. For this study, loss estimates were derived from a flood risk model that combined hazard data provided by KatRisk and a Canadian exposure dataset (Morin et al. 2025). This section describes key elements of the flood risk modeling methodology.

Canada's flood‐risk data ecosystem is expanding rapidly. However, it still lacks a unified, national, household‐level flood risk dataset comparable to publicly accessible, high‐resolution resources available in some other countries such as the United States. FEMA's National Flood Hazard Layer, the United Kingdom's Flood Hub, Greece's ERMIS‐F, or commercial tools such as the US‐based First Street Foundation's Flood Factor. Canadian provinces and territories do produce regulatory flood maps for high‐risk areas, but geographic coverage remains uneven, methodology differs, and public accessibility varies considerably. Private commercial flood‐risk providers also operate in Canada, but none provide full household‐level, publicly accessible maps. Their products are mostly licensed to insurers and governments and used internally for underwriting and catastrophe modeling.

The flood‐risk data used in this article are constructed by combining the best available high‐resolution, near‐nationally comprehensive datasets on property locations and their characteristics in Canada, with flood modeling from a commercial data provider. More specifically, the Canadian exposure dataset is built with data from the 2021 Census (Statistics Canada), DMTI Spatial's CanMap Address Points, and Verisk Canada's (formerly Opta) Aggregate Exposure Information. For each dissemination area^1^ in the 2021 Census, the number of single‐detached houses, semi‐detached houses, row houses, and apartments or flats in a duplex (excluding multidwelling units and mobile homes) were extracted and assigned latitude and longitude coordinates with rooftop‐level precision using DMTI Spatial's CanMap Address Points. Property characteristics come from Verisk Canada's Aggregate Exposure Information datasets, which provide descriptive statistics on key property characteristics aggregated at the dissemination block (DB)^2^ (statistics are aggregated at the dissemination area level when the number of dwellings in a block is insufficient). Reconstruction costs, number of floors, and basement presence were randomly assigned to buildings within each DB to match the descriptive statistics. The dataset comprised 9.8 million dwellings over Canada.

**FIGURE 1:**
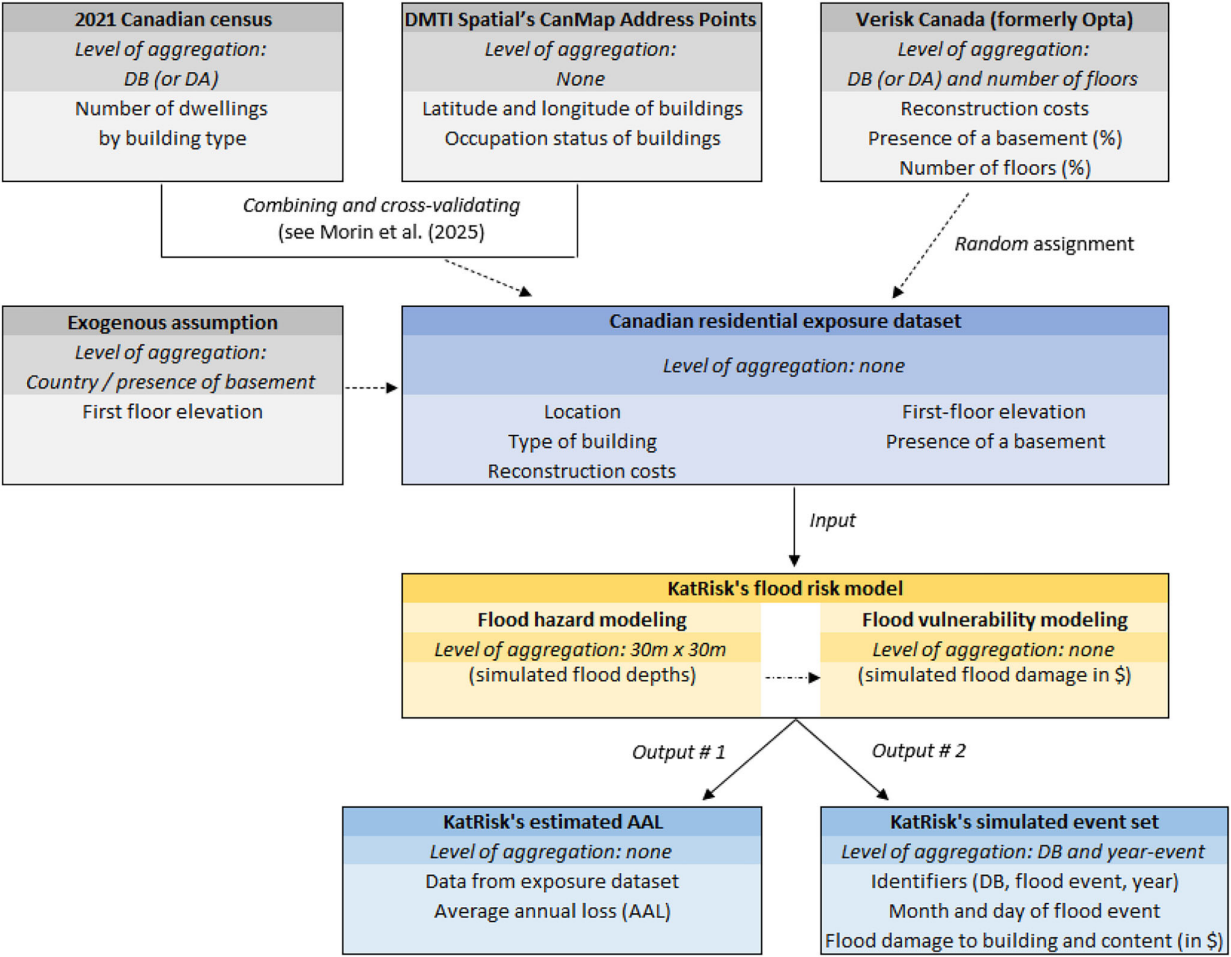
Data flow diagram for the Canadian flood risk model. DB, dissemination block.

The exposure dataset was then provided to KatRisk to assess the flood hazard and vulnerability. The Canadian flood model from KatRisk covers pluvial and fluvial flooding at 30 m resolution for the 10 provinces. It has been validated with regulatory flood maps by Public Safety Canada as part of the National Task Force on Flood Insurance and Relocation (Public Safety Canada 2022). The model includes basic flood defense data but may lack specific flood protection works in certain areas. The vulnerability of individual properties is derived from data by the US Army Corps of Engineers (USACE) and from the US Federal Insurance Administration. More details about KatRisk's flood hazard and vulnerability models can be found in KatRisk (2022). Figure 1 offers a schematic representation of the data flow involved in creating the Canadian residential exposure dataset and running KatRisk's flood risk model. The output provided by KatRisk comprises the average annual loss (AAL) for each of the 9.8 million dwellings in addition to an “event set” made of 700,000 synthetic dated flood events occurring over 50,000 simulated years. The event set is presented as a set of tables detailing the simulated loss for each event aggregated per DB. The event set reveals that the total average annual flood loss in Canada is $1.4 billion, with a 1% probability of losses exceeding $13 billion in any given year.

We acknowledge that our flood estimates may fall short of fully capturing property‐level risk,^3^ in part because some characteristics are randomly attributed. However, this source of error is limited, as the random assignment occurs essentially among neighboring properties, which typically share similar structural features. Indeed, Moralı and Yılmaz (2022), Ismail (2006), and many others document strong spatial correlations in structural property characteristics. Moreover, although we rely on local data, our objective is to produce Canada‐wide estimates. In this context, local over‐ and under‐estimations are expected to largely offset one another, so any remaining “noise” averages out in the aggregate national results.

### 3.2 Calculation of Insurance Premium

Property‐level flood losses derived from KatRisk's event set reflect the cost to rebuild or repair damaged property and replace content. The ground‐up loss is then adjusted by incorporating a deductible and policy limit to compute the insured loss, or the amount that is reimbursed to a flood victim by an insurance company. For example, if a flood causes $250,000 damage to a residential home, with a deductible of $25,000, the insured loss is $225,000. The actuarially fair‐ or fully risk‐based insurance premium is calculated in a four‐step process. First, the level of deductible and coverage limit is assumed to be the same for all homeowners throughout the country. Second, the insured loss associated with each flood event and each residential property is assigned from the disaggregated event set (see Appendix A for more details). Third, insured losses are aggregated by year and property to calculate the average annual insured loss (AAIL). The AAIL thus represents the average amount of loss covered by a policy that an insurer should compensate to a policyholder over a year.

In the fourth and last step, the premium should reflect additional charges associated with sales (e.g., commissions), insurer administrative costs (e.g., human resources), reinsurance, profit margins, and coverage for additional living expenses (e.g., hotel stays). These items are added to the AAIL to obtain a sensible estimate of the insurance premium (Open Actuarial Community 2020; Louaas and Picard 2021). To account for these additional charges, the authors added 80% of the AAIL on the basis of findings from previous studies (e.g., Davis et al. 2018). The next section develops metrics that were developed to measure the objectives of affordability, compensation adequacy, and participation to enable a better understanding of the trade‐offs involved for Canadian decision‐makers.

## 4 Insurance Design Analysis

### 4.1 Baseline Scenario Using a Risk‐Based Premium

To understand how different design choices for the CFIP affect outcomes, a risk‐based premium was used to develop a baseline scenario—an actuarially fair distribution of pricing before any policy interventions to improve affordability, availability, or compensation adequacy are considered. This choice reflects the fact that premium subsidies should disproportionally benefit homeowners located in high‐risk areas given that high level of expected losses translates into high insurance premium.

Table 1 illustrates the amount and concentration of flood risk in Canada in terms of AAL as estimated by the flood risk model. Note that the AAL is invariant to the design characteristics of the Canadian flood insurance system. On average, flood losses are projected to cause $1.4 billion in damage per year. The distribution of these losses is very uneven across the homeowners.

**TABLE 1:** Amount and concentration of flood losses in Canada by risk category.

Risk category	Pctl	AAL		Number of households	AAL (M$)	% household	% AAL	Mean premium ($)
		Min. ($)	Max. ($)					
Very‐low risk	[Min. to 50]	0	31	4,903,579	64.6	50	5	23.7
Low risk	[50–90]	31	60	3,923,444	231.0	40	16	106.0
Moderate risk	[90–95]	60	293	490,423	87.8	5	6	322.2
High risk	[95–99]	293	3498	392,307	448.8	4	32	2059.4
Very‐high risk	[99 to max.]	3498	41,815	98,079	580.8	1	41	10,659.0
**Canada‐wide**				**9,807,832**	**1413**	**100**	**100**	**259.3**

Abbreviation: AAL, average annual loss.

Indeed, most households are at low or very‐low risk of flooding. These two categories account for 90% of the 9.8 million Canadian residential homeowners but account for only about 21% of flood losses. Households at moderate risk of flooding are those that fall between the 90th and 95th percentile of the AAL distribution. The relative importance of flood risk for this category (6% of AAL) is similar to its relative size (5% of households). Conversely, although only 5% of Canadian households are at high (or very‐high) risk of flooding, these residential properties account for more than 70% of the expected annual flood losses. The top 1% of the AAL distribution, the very‐high‐risk homeowners, comprises more than 40% of flood risk. The concentration of flood risk in Canada confirms the challenge of adverse selection and the need to incorporate design elements to diversify risk in ways that ensure adequate participation.

The last column of Table 1 details the average insurance premium in the baseline scenario, with flood losses being totally insured (no deductible, no policy limit), full participation, and no subsidies. The cost of insurance increases sharply with the risk categories. Homeowners at moderate risk can be expected to pay a few hundred dollars to be fully insured, whereas high‐risk homeowners must anticipate paying a few thousand dollars for the same protection. It is worth noting that an insurance design with a flat pricing approach would cost every property owner about $260, which is more than the actuarially fair premium for 90% of homeowners. This finding suggests that perceptions of the utility of purchase among low‐ and moderate‐risk areas could be insufficient for demand in a flat pricing model.

### 4.2 Affordability

Flood insurance affordability can be assessed using several different metrics that compare the price of the premium‐to‐income measure (FEMA 2018; Public Safety Canada 2022). Hudson (2018) distinguishes three groups of affordability metrics based on the ratio of premium to disposable income, disposable income above a poverty line (residual income), and the housing cost burden, respectively. On the basis of data availability, flood insurance affordability in Canada was measured by ratios of premiums to disposable income and more precisely by the ratio of premium on after‐tax income. Median after‐tax income data are available from the 2021 Census at the dissemination area level. We further disaggregated these data to estimate household‐level income by assuming that the after‐tax income of each household within a dissemination area is proportional to the reconstruction cost of its property relative to that of the average property in the area. This helps preserve the strong observed positive correlation between income and property value (e.g., Maoh and Abdo 2025), which is particularly important in neighborhoods with a large observed dispersion in reconstruction costs.^4^

To demonstrate how premiums influence affordability, five categories were developed (see Table 2). Premiums above 1.8% of after‐tax income were deemed unaffordable. This threshold is based on the United Kingdom's Flood Re where the 1.8% median income to premium ratio is used to determine the maximum premium (Supplementary Material from Hudson et al. 2019).

**TABLE 2:** Number of households (*n*) and size of flood risk (average annual loss [AAL]) by risk and affordability categories in the base case scenario.

		Ratio of premium to after‐tax income					
		[Min. to 0.5%]	[0.5%–1.0%]	[1.05–1.8%]	[1.8%–3.0%]	[3.0% to max.]	Total
		Very reasonable	Reasonable	Neutral	Expensive	Very expensive	
**Percentiles of average annual loss (AAL)**	**Very‐low risk** (≤50th pctl)	*n* = 4,903,579	*n* = 0	*n* = 0	*n* = 0	*n* = 0	*n* = 4,903,579
		AAL = $65 M	AAL = $0 M	AAL = $0 M	AAL = $0 M	AAL = $0 M	AAL = $65 M
	**Low risk** (50th–90th pctl)	*n* = 3,914,608	*n* = 8750	*n* = 86	*n* = 0	*n* = 0	*n* = 3,923,444
		AAL = $230 M	AAL = $1 M	AAL = $0 M	AAL = $0 M	AAL = $0 M	AAL = $231 M
	**Moderate risk** (90th–95th pctl)	*n* = 366,045	*n* = 118,691	*n* = 5519	*n* = 167	*n* = 1	*n* = 490,423
		AAL = $61 M	AAL = $25 M	AAL = $1 M	AAL = $0 M	AAL = $0 M	AAL = $88 M
	**High risk** (95th–99th pctl)	*n* = 11,166	*n* = 76,808	*n* = 93,149	*n* = 73,190	*n* = 137,994	*n* = 392,307
		AAL = $4 M	AAL = $31 M	AAL = $56 M	AAL = $73 M	AAL = $286 M	AAL = $449 M
	**Very‐high risk** (>99th pctl)	*n* = 0	*n* = 0	*n* = 0	*n* = 45	*n* = 98,034	*n* = 98,079
		AAL = $0 M	AAL = $0 M	AAL = $0 M	AAL = $0 M	AAL = $581 M	AAL = $581 M
	**Total**	*n* = 9,195,398	*n* = 204,249	*n* = 98,754	*n* = 73,402	*n* = 236,029	***n* =** **9,807,832**
		AAL = $360 M	AAL = $57 M	AAL = $57 M	AAL = $73 M	AAL = $866 M	**AAL = $1413 M**

Under this scenario, more than 300,000 Canadian households will pay premiums that exceed 1.8% of after‐tax income (shaded in light green in Table 2). To generate a measure of affordability, the total number of households with affordable (very reasonable and reasonable columns in Table 2) and unaffordable (expensive and very expensive columns in Table 2) premiums were divided by the total number of insured households (see the following equations):

(C1.1) $$\begin{array}{ccc} & & \mathrm{Reasonable}\;\mathrm{premium}\\ & & =\frac{\mathrm{Number}\;\mathrm{insured}\;\mathrm{households}\;\mathrm{with}\;\mathrm{affordable}\;\mathrm{premium}}{\mathrm{Total}\;\mathrm{number}\;\mathrm{of}\;\mathrm{insured}\;\mathrm{households}} \end{array}$$

(C1.2) $$\begin{array}{ccc} & & \mathrm{Expensive}\;\mathrm{premium}\;\\ & & =\frac{\mathrm{Number}\;\mathrm{insured}\;\mathrm{households}\;\mathrm{with}\;\mathrm{unaffordable}\;\mathrm{premium}}{\mathrm{Total}\;\mathrm{number}\;\mathrm{of}\;\mathrm{insured}\;\mathrm{households}} \end{array}$$

Flood insurance is affordable for over 95% of households (shaded blue in Table 2) including some properties at moderate or even high risk of flooding, and unaffordable for 3.2% of households. The mean premium‐to‐income ratio (C1.3) for the subset of properties at high (and very‐high) risk of flooding is 5.4%, which exceeds the 1.8% unaffordability threshold. This result indicates that fully risk‐based premiums would be unaffordable in high‐risk areas.

(C1.3) $$\begin{array}{ccc} & & \mathrm{Mean}\;\mathrm{PR}-\mathrm{INC}\;\mathrm{ratio}\;\mathrm{high}\;\mathrm{risk}\\ & & =\frac{\mathrm{Sum}\;\mathrm{premiumtoincome}\;\mathrm{ratio}\;\mathrm{of}\;\mathrm{insured}\;\mathrm{household}\;\mathrm{at}\;\left(\mathrm{very}\right)\;\mathrm{high}\;\mathrm{risk}}{\mathrm{Total}\;\mathrm{number}\;\mathrm{of}\;\mathrm{insured}\;\mathrm{households}\;\mathrm{at}\;\left(\mathrm{very}\right)\;\mathrm{high}\;\mathrm{risk}} \end{array}$$

**FIGURE 2:**
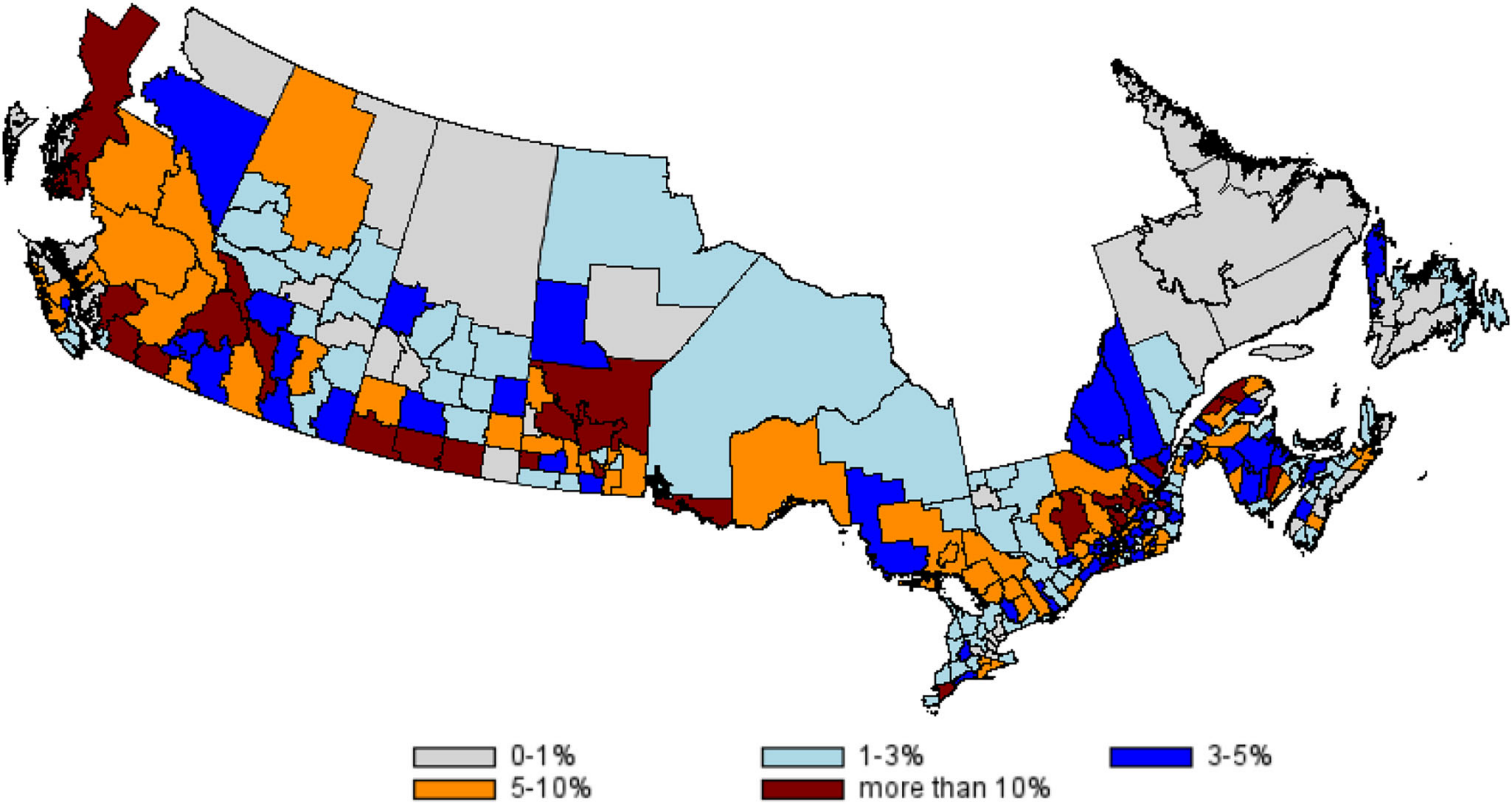
Prevalence of expensive (and very expensive) flood insurance by census division in the base case scenario.

Although AAL is concentrated in high‐risk areas, Figure 2 shows that unaffordable premiums are distributed throughout the country. More than 10% of homeowners will find flood insurance expensive or very expensive (regions in red) in 23 census divisions spread over seven provinces. In 86 census divisions across eight provinces, insurance will be unaffordable for 5% of property owners. When comparing the three most populous cities, there is also a diverse range of unaffordability—lowest in Toronto (0.64%), followed by Greater Vancouver (2.31%) and Montreal (3.26%).

To improve affordability, governments often deploy financial assistance to offset the cost. Two premium subsidy schemes were evaluated for their impact on affordability. The first used a premium‐on‐income measure of affordability that progressively increases as the premium‐on‐income ratios rise (Table 3). A first subsidy level reimburses households for 25% of the portion of premium between 1.5% and 2% of their after‐tax household income. A second level compensates for 50% of the premium between 2% and 2.5% of their household income. A third level returns 75% of the cost of insurance that falls between 2.5% and 3% of the income. The last level subsidizes 90% of the portion of the premium deemed very expensive. Table 3 summarizes the impact of this subsidy for a household earning $60,000 in after‐tax income per year with a $2000 flood insurance premium (5% of after‐tax income).

**TABLE 3:** Parametrization of a hypothesized government subsidy scheme with working example based on a $60,000 after‐tax income family facing a $2000 flood insurance premium.

	Premium‐on‐income ratio			Example ($2000 premium and $60,000 after‐tax income)			
Tranche	Min. (%)	Max. (%)	Subsidies (%)	Thresholds($60k income)	Tranche of premium ($)	Amount of subsidies ($)	Net cost of insurance ($)
1	0	1.5	0	$900	900	0	900
2	1.50	2.0	25	$1200	300	75	225
3	2.00	2.5	50	$1500	300	150	150
4	2.50	3.0	75	$1800	300	225	75
5	3.00	∞	90	Over $1800	200	180	20
					**Premium** (before subsidies)	**Amount of subsidies**	**Net premium** (after subsidies)
					2000	630	1370

The second subsidy scheme enforces a maximum premium fixed at 3% of the median national after‐tax household income ($73,000). Households would pay a risk‐based premium up to $2190, but any amount exceeding that threshold is compensated by the government. Table 4 reveals the impact of the subsidies and caps under the baseline scenario (i.e., full participation and complete coverage). The total cost for government is over $1 billion each year for the two schemes.

**TABLE 4:** Impact of premium caps and subsidies on affordability, compensation adequacy, and participation.

	Affordability(among insured households)		
	C1.1	C1.2	C1.3
Scenario	Reasonable premium (%)	Expensive premium (%)	Mean PR‐INC ratio(high risk) (%)
Base case	95.8	3.2	5.4
Progressive subsidies	95.8	3.1	2.1
Premium cap	95.8	3.0	2.3

The subsidy schemes clearly improve affordability with a decrease in the mean‐premium income ratio among high‐risk properties below 3%, whereas the ratio of reasonable to expensive premiums remains the same.

This section revealed that a risk‐based premium will limit affordability in high‐risk areas distributed throughout Canada and justified some form of subsidization to offset these costs as a means of encouraging market participation. The next section explores how adjustments to the risk‐based premium through deductibles and limits influence the objective of adequate insurance compensation.

### 4.3 Compensation Adequacy

Adequate and predictable financial compensation is a major objective of Canada's flood insurance design (Public Safety Canada 2022). Demand and market participation might not be sufficient if households do not perceive compensation is adequate relative to the cost of premiums. Analysis of affordability using a risk‐based premium reveals that property owners in high‐risk areas are unlikely to afford comprehensive coverage, meaning they could purchase coverages with partial or smaller claim payments.

Adequate compensation was assessed using two criteria. The residual risk criterion (C2.1) investigated the extent to which coverage is comprehensive at a national scale. The baseline scenario entails no residual risk as all flood losses are compensated through insurance, whereas the complete absence of flood insurance would translate into a residual risk of one. Lower values for residual risk indicate higher rates of adequate compensation:

(C2.1) $$\mathrm{Residual}\;\mathrm{risk}=1-\frac{\mathrm{sum}\;\mathrm{of}\;\mathrm{AAIL}}{\mathrm{sum}\;\mathrm{of}\;\mathrm{AAL}}$$

The second criterion measured the mean proportion of uninsured loss among high‐risk areas (C2.2), where more expensive premiums could lead policyholders to purchase less coverage, leading to more uninsured flood losses. In the baseline scenario of full insurance, the numerator of this criterion is zero as all losses are compensated through insurance, meaning lower values are a strong indicator for adequate compensation:

(C2.2) $$\begin{array}{ccc} & & \mathrm{Mean}\;\mathrm{uninsured}\;\mathrm{loss}\;\mathrm{high}\;\mathrm{risk}\\ & & =\frac{\mathrm{sum}\;\mathrm{of}\;\left(1-\mathrm{AAIL}/\mathrm{AAL}\right)\;\mathrm{over}\;\left(\mathrm{very}\right)\;\mathrm{high}\;\mathrm{risk}\;\mathrm{households}}{\mathrm{Total}\;\mathrm{number}\;\mathrm{of}\;\mathrm{households}\;\mathrm{at}\;\left(\mathrm{very}\right)\;\mathrm{high}\;\mathrm{risk}} \end{array}$$

Deductibles and policy limits are the main design elements that could influence adequate compensation. These tools are used to promote affordability throughout the pool of insureds by offsetting the cost of premiums through one‐time deductible payments in the event of a claim or limits on the total amount of coverage that a household can access per event. Table 5 illustrates the impact of eight combinations of deductibles and limits on compensation adequacy and affordability.

**TABLE 5:** Impacts of deductibles and policy limits on affordability and compensation adequacy.

			Affordability (among insured households)			Compensation adequacy	
			C1.1	C1.2	C1.3	C2.1	C2.2
Scenario	Deductible ($)	Limit ($)	Reasonable premium (%)	Expensive premium (%)	Mean PR‐INC ratio (high risk) (%)	Residual risk (%)	Mean uninsured loss (high risk) (%)
Base case	0	No limit	95.8	3.2	5.4	0.0	0.0
2	1000	No limit	96.0	3.1	5.3	5.3	3.3
3	5000	No limit	96.2	2.9	5.1	15.4	12.5
4	10,000	No limit	96.5	2.8	4.8	23.2	21.1
5	25,000	No limit	96.9	2.4	4.2	37.7	38.8
6	1000	300,000	96.0	3.1	4.8	13.0	7.5
7	5000	300,000	96.3	2.9	4.6	23.1	16.7
8	10,000	300,000	96.5	2.7	4.3	30.9	25.3
9	25,000	300,000	97.0	2.4	3.7	45.4	43.0

Adding deductibles and limits improves affordability. The percentage of households exposed to expensive premiums drops from 3.2 with a risk‐based premium to between 2.9 with a deductible of $5000 and 2.4 with a deductible of $25,000. Combining a deductible with a $300,000 limit increases affordability with 97% of households now under the 3% premium‐to‐income ratio. Similar improvements in affordability are found for high‐risk households as demonstrated by a reduction in the mean premium‐to‐income ratio from 5.4% for the baseline scenario to 3.7% with a $25,000 deductible and $300,000 limit.

Although deductibles and limits improve affordability, they have the opposite effect on adequate compensation. In fact, uninsured flood losses, as measured by residual risk (C2.1), grow as the deductible increases or as a policy limit is added to the insurance design. More than a third of all flood losses become uninsured when a $25,000 deductible is imposed, and that percentage increases significantly to 45.4% when a limit is combined with the $25,000 deductible. The mean fraction of uninsured losses in high‐risk areas (C2.2) follows the same pattern as the residual risk metric.

### 4.4 Participation

The baseline scenario assumes full participation, but this is unlikely in Canada. Compulsory participation would face political opposition as most properties in Canada are in low‐ and moderate‐risk zones that do not necessarily need coverage (Thistlethwaite and Henstra 2024). Moreover, provinces are responsible for regulating insurer market conduct, and they are unlikely to implement mandatory purchase unanimously, leaving gaps in participation.

To measure how participation influences the other variables, three scenarios were developed as the baseline assumes full participation. First, each homeowner was assumed to have an 80% probability to buy insurance regardless of the price. The 80% was uniformly distributed among the risk and the affordability categories. The second take‐up scenario excluded very‐high‐risk areas from insurance coverage. The rationale was that the magnitude of flood risk and cost of coverage for the top 1% of the AAL distribution are so significant that demand will be too low, and governments would be unwilling to subsidize and absorb the financial instability associated with a highly volatile market. The last take‐up scenario assumed that the decision to purchase flood insurance is driven solely by the price of insurance. In this scenario, households do not buy insurance when the premium is very expensive. Each of these scenarios is described by comparing the total number of insured households at very‐high risk to the total number of households at very‐high risk:

(C3) $$\begin{array}{ccc} & & \mathrm{HighRisk}\;\mathrm{takeup}\\ & & =\frac{\mathrm{Total}\;\mathrm{number}\;\mathrm{of}\;\mathrm{insured}\;\mathrm{households}\;\mathrm{at}\;\left(\mathrm{very}\right)\;\mathrm{high}\;\mathrm{risk}}{\mathrm{Total}\;\mathrm{number}\;\mathrm{of}\;\mathrm{households}\;\mathrm{at}\;\left(\mathrm{very}\right)\;\mathrm{high}\;\mathrm{risk}} \end{array}$$

Table 6 explores how the different participation rates influence outcomes for affordability and compensation adequacy. If the government decides to exclude the top 1% of flood exposure, affordability improves, as is evident in a premium‐to‐income ratio below 3%. But lower participation also leads to less adequate compensation with increases in residual risk and the mean uninsured losses. This result is consistent with existing research that found that achieving high rates of participation is necessary to achieve adequate coverage, but those in high‐risk areas will struggle with affordability.

**TABLE 6:** Impacts of participation on affordability, compensation adequacy, and participation.

	Affordability (among insured households)			Compensation adequacy		Participation
	C1.1	C1.2	C1.3	C2.1	C2.2	C3
Scenario	Reasonable premium (%)	Expensive premium (%)	Mean PR‐INC ratio (high risk) (%)	Residual risk (%)	Mean uninsured loss (high risk) (%)	Take‐up rate (high risk) (%)
Base case	95.8	3.2	5.4	0.0	0.0	100.0
80% random	95.8	3.2	5.4	20.0	20.0	80.0
Excl. very‐high risk	96.8	2.2	3.0	41.1	20.0	80.0
Excl. very expensive	98.2	0.8	1.4	61.3	48.1	51.9

## 5 Insurance Design and Public Policy Objectives

This section creates a set of insurance design scenarios to explore how they influence the public policy objectives of affordability, adequate compensation, and participation. Nine mixes of deductibles and limits (Table 5) were compared to four participation schemes (Table 6). In addition, the public cost of the caps and subsidies was included in the analysis. These combinations created 108 distinct scenarios that revealed outcomes for affordability, compensation, and participation. Table 7 illustrates the performance of nine selected scenarios (see Appendix B for details on all of the 108 scenarios).

**TABLE 7:** Performance metrics associated with the policy objectives for nine selected scenarios combining deductibles, limits, participation schemes, and subsidy schemes.

	Affordability (among insured households)			Compensation adequacy		Participation	
	C1.1	C1.2	C1.3	C2.1	C2.2	C3
Scenario	Reasonable premium (%)	Expensive premium (%)	Mean PR‐INC ratio (high risk) (%)	Residual risk (%)	Mean uninsured loss (high risk) (%)	Take‐up rate (high risk) (%)	Cost of public assistance ($M)
*[Base]—No deduct*. *No subs*. *Full part*.	95.8	3.2	5.4	0.0	0.0	100.0	0.0
*[2]—$10k deduct*. *No subs*. *Full part*.	96.5	2.8	4.8	23.2	21.1	100.0	0.0
*[3]—$25k deduct*. *Progr. subs*. *Full part*.	96.9	2.4	1.7	37.7	38.8	100.0	829.7
*[4]—$10k deduct*. *Progr. subs*. *Exc. very‐high risk*	97.5	1.7	1.5	61.9	39.8	80.0	229.8
*[5]—$10k deduct*. *Premium caps*. *Exc. very expen*.	97.8	1.4	1.4	51.7	44.2	74.1	485.6
*[6]—$10k deduct*. *Progr. subs*. *Exc. very expen*.	97.2	2.0	1.6	53.0	34.5	85.8	375.2
*[7]—$25k deduct*. *No subs*. *Exc. very expen*.	98.8	0.5	1.0	84.9	69.6	61.8	0.0
*[8]—$25k deduct*. *Progr. Subs*. *Exc. very expen*.	97.5	1.8	1.4	62.4	49.3	87.9	340.8
*[9]—$10k deduct. $300k limit* *Progr. subs*. *Excl. very expens*.	97.1	2.1	1.6	50.9	34.7	88.5	402.9

On the basis of these scenarios, it is evident that compensation adequacy declines as measures to improve premium affordability are included, such as deductibles, policy limits, and exclusions. Scenario 7 offers the most extreme example of this relationship by achieving the most optimal outcome for affordability with only 0.5% of policyholders paying expensive premiums and a premium‐to‐income ratio at 1.0% for the high risk. Excluding very expensive households, however, leads to a suboptimal level of residual risk (84.9%) and mean uninsured loss (69.6%). Scenario 2 attempts to moderate this trade‐off with full participation, limiting residual risk and mean uninsured loss to 23.2% and 21.1%, respectively, but affordability remains high with 2.8% paying expensive premiums and a premium‐to‐income ratio of 4.8% in high‐risk areas, likely because the full cost of flood liability in high‐risk areas is distributed across all households. Adding subsidies along with a $25k deductible (Scenario 3) improves affordability with a premium‐to‐income ratio declining 3.7% from the baseline residual risk and uninsured loss increasing to 37.7% and 38.8%, respectively. There is also evidence from Scenario 7 that subsidies moderate the impact of deductibles and limits on compensation adequacy. This scenario does not include a subsidy and has the highest levels of residual risk and uninsured loss.

In addition to increasing residual risk, deductibles, limits, and market exclusions decrease the participation rate. Scenario 7 offers a useful example on the impact of exclusions and deductibles by improving the premium‐to‐income ratio to 1.0% in high‐risk areas. But participation is the lowest among all the scenarios (61.8%) due to the exclusion. Like compensation adequacy, subsidies do moderate the impact of the exclusions. Scenario 8 includes a progressive subsidy along with a high‐risk market exclusion at an expense to government of $340.8 million that improves the participation rate by 26% compared to Scenario 7 while sustaining affordability (1.4% premium‐to‐income ratio for high risk).

Among the scenarios without compulsory coverage, Scenario 9 achieves the best balance between affordability, coverage adequacy, and participation through the introduction of a $300,000 limit on coverage along with excluding very expensive policies. Compared to the baseline, Scenario 9 reduces those households paying expensive premiums by 1.1% and the premium‐to‐income ratio by 3.8%, while keeping residual risk at about 50% and uninsured loss below 35%, and achieves the highest participation rate outside of compulsory purchase (88.5%). Given the positive effect of introducing a limit on the participation rate, Scenario 9 displays a higher cost to government ($402.9 M) relative to a similar scenario without such a limit ($375.2 M in Scenario 6). Although Scenario 9 achieves the best balance among the main objectives, it is still weaker compared to the compulsory purchase models due to higher levels of residual risk and uninsured loss and a lower rate of participation.

To improve these measures, Scenario 10 of Table 8 identifies a subsidy, deductible, and limit scheme that reduces the number of high‐risk households excluded. Increasing the rate of subsidization between premium‐to‐income categories as outlined in Section 4.2 (Table 3), combined with a premium cap, represents an ambitious means of lowering the cost to high‐risk homeowners and expanding participation. Subsidies were increased from 25% to 60% for those with a premium‐to‐income ratio of 1.5%–2.0%, 50%–70% for those between 2.0% and 2.5%, 75%–80% for those between 2.5% and 3.0%, and held at 90% for those above 3%. As this subsidy scheme increases the financial burden on government, the total amount was limited to $10,000 per household.

**TABLE 8:** Calibrated scenario (number 10) to maximize alignment with insurance objectives.

	Affordability (among insured households)			Compensation adequacy		Participation	
	C1.1	C1.2	C1.3	C2.1	C2.2	C3	
Scenario	Reasonable premium (%)	Expensive premium (%)	Mean PR‐INC ratio (high risk) (%)	Residual risk (%)	Mean uninsured loss (high risk) (%)	Take‐up rate (high risk) (%)	Cost of public assistance ($M)
*[3]—$25k deduct*. *Progr. subs*. *Full part*.	96.9	2.4	1.7	37.7	38.8	100.0	829.7
*[9]—$10k deduct. $300k limit* *Progr. Subs*. *Excl. very expens*.	97.1	2.1	1.6	50.9	34.7	88.5	402.9
*[10]—$10k deduct*. *$300k limit* *Subsidies + cap* *Exc. Pr‐inc ratio above 3.5%*	96.6	2.2	1.6	37.3	27.6	97.2	733.6

Analysis of Scenario 10 confirms the relationships observed in Table 7 that subsidies moderate the impacts of deductibles and limits on residual risk and exclusions on affordability, compensation adequacy, and market participation. Increasing the subsidy significantly for the first premium‐to‐income segment and more gradually for more expensive ones represents an efficient allocation of resources that limits burden on government while improving affordability to increase participation among high‐risk households. Compared to Scenario 9, Scenario 10 reduces residual risk by 13.6% (37.3%), and the take‐up rate in Scenario 10 is 8.7% (97.2%) higher than Scenario 9. This improvement comes with a higher cost of caps and subsidies for governments but remains $96.1 M lower than under the full participation scheme (Scenario 3). Achieving this balance still requires an exclusion of those with a premium‐to‐income ratio that exceeds 3.5%, but in this scheme, this amounts to 13,600 households, whereas more than 56,329 were excluded in Scenario 9. Even though a large amount of losses associated with high‐risk areas are being excluded, the mean uninsured loss among high‐risk households improves to 27.6%. This gap in coverage is the smallest among all scenarios and even surpasses Scenario 3 where participation is compulsory.

**FIGURE 3:**
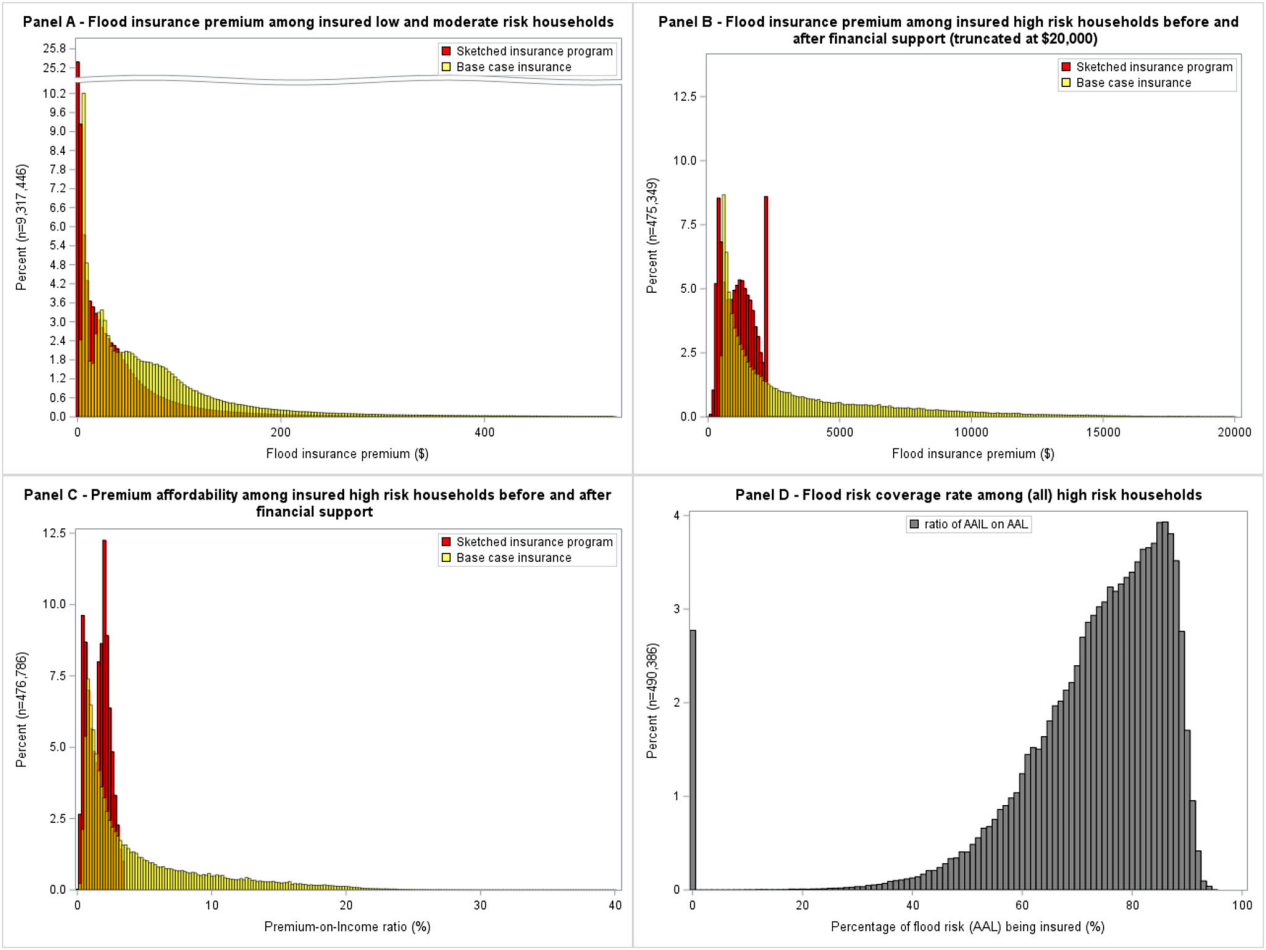
Cost of flood insurance for low‐ and medium‐risk homeowners (Panel A) and high‐risk homeowners (Panel B) and affordability of insurance (Panel C) and compensation adequacy (Panel D) in high‐risk location. AAL, average annual loss.

Figure 3 demonstrates some of the benefits of Scenario 10. Panel A shows how the number of households paying cheaper premiums increases with the introduction of deductibles and limits as the liability for paying for losses is allocated more toward those in higher risk segments. Indeed, the premium for those at (very) low and moderate risk of flooding drops from $74 in the baseline scenario to $32.70 in Scenario 10 even though these households do not meet the premium‐to‐income ratio to qualify for caps or subsidies (>1.5%). Affordability also improves for high‐risk owners outside of those excluded (Panel B). The mean (median) premium decreases in Scenario 10 to $1168 ($1138) from $3460 ($1956) in the baseline scenario, leading to a significant increase in the percentage of affordable premiums in high‐risk areas (Panel C). Scenario 10 also improves compensation adequacy among high‐risk property owners with policies accounting for, on average (median), 74.5% (76.6%) of flood damage (Panel D). The policy exclusion is evident in the panel as well, with 2.77% of high‐risk households receiving no coverage.

The existing literature shows that keeping insurance prices proportional to underlying risk is central to limiting adverse selection and moral hazard (Boudreault and Ojeda 2022; Bradt et al. 2021). Reflecting this, Scenarios 9 and 10 exhibit rank correlation coefficients among high‐risk insured households of 0.865 and 0.966, respectively, demonstrating that affordability gains can be achieved without significantly eroding the link between insurance price and underlying insured risk. This result suggests that it is possible to design flood insurance programs in ways that limit the potential impacts of moral hazard on household decisions and behavior.

This section revealed the relationship between the design elements of deductibles, limits, subsidies, premium caps, and exclusions on the policy objectives of affordability, compensation adequacy, and market participation. Deductibles and limits do improve affordability by lowering the premium but decrease compensation and participation. Subsidies moderate these impacts while improving affordability as evident in Scenario 7, which does not include a subsidy and has the most suboptimal performance. Scenario 9 revealed how the introduction of a $300,000 limit along with a $10,000 deductible, subsidies, and high‐risk exclusion managed to balance the trade‐off between affordability, compensation adequacy, and market participation. This design lowered the costs of insuring high‐risk areas but managed to sustain a higher level of participation than other scenarios with a subsidy reducing the cost of premiums. Scenario 10 built on these findings by calibrating the design to establish a better balance between the public policy objectives by increasing the rate of the subsidy across risk segments, capping premiums at 3% of the median national after‐tax household income, and placing a total limit of $10,000 for each subsidy per household. This scenario increased market participation more than every other non‐compulsory scenario and limited the market exclusion to 13,600 households in the highest risk segment. This analysis reveals that an aggressive and progressive subsidy, a small deductible, a limit, and a high‐risk exclusion are an optimal design to balance affordability and compensation adequation in the CFIP.

## 6 Conclusion

This article used a novel Canadian flood risk model to evaluate how the design of the CFIP could maximize its public benefit by meeting the objectives of affordability, adequate compensation, and market participation. This balance is elusive given that premium affordability in high‐risk areas requires deductibles, policy limits, and exclusions that reduce the utility of purchasing coverage, thereby leading to less demand and an insufficient rate of market participation. Subsidies can improve demand by lowering the cost of premiums but place a significant financial burden on governments and disrupt risk‐based price signals designed to motivate risk reduction.

To identify an optimal design, the article developed a baseline insurance scenario using fully risk‐based premiums and then constructed metrics for affordability, adequate coverage, and participation by measuring how design elements achieve each compared to the baseline. Findings from the impact of deductibles, limits, coverage exclusions, and subsidies were then combined into an analysis of nine different design scenarios. These scenarios revealed that as deductibles, limits, and exclusions were used to improve affordability in high‐risk areas by distributing the burden for the financial liability, compensation adequacy and market participation decreased. Subsidies in the form of direct payments and premium caps did moderate these impacts on high‐risk households with evidence that compensation and participation improved. To confirm this assumption, an additional scenario was developed that increased the magnitude of the subsidy, capped premiums, and limited subsidies to $10,000 per household. Based on these findings, to maximize the public benefits of flood insurance in Canada, policymakers should adopt a progressive subsidy along with premium caps, a moderate deductible, and a limit on coverage in addition to a market exclusion for the highest risk households (13,600).

It is important to note several data gaps that limit this analysis and justify future research. As discussed in Morin et al. (2025), flood modeling in Canada remains in a nascent phase, resulting in uncertainty that requires validation using historical and government data sources. As these data were used as the underlying input for analyzing flood insurance design in Canada, those uncertainties apply to this article as well. Second, there are assumptions involved in determining metrics for affordability, adequate compensation, and market participation that require further investigation with policymakers and insurers or diagnosis through household surveys. For example, it is worth engaging with stakeholders to determine if a 1.8% premium‐to‐income affordability threshold using disposable income is more appropriate than total income. If total income was used, coverage could be more affordable since the premium will be lower relative to total income than disposable income. Third, the analysis did not compare individual relationships between design elements and policy objectives but rather grouped them into scenarios. More research could be done to determine if adjusting one design element alone had more of an impact on these objectives than others. Lastly, the article did not explore the impact of insurance design elements on risk reduction, which is often considered a benefit of insurance systems over public assistance. Determining if a premium price is sufficient to incentivize a household or community to invest in risk reduction remains uncertain and requires further scrutiny to be included in the scenario analysis.

The CFIP will likely have optional participation from homeowners, and to mitigate the effects of adverse selection, a public–private arrangement should aim at maximizing participation while preserving risk‐adjusted premiums as much as possible. Although caps and subsidies can easily improve affordability, they may also trigger adverse selection if the price signal is mostly attenuated (e.g., a single premium cap). Deductibles and limits also make insurance more affordable and can be calibrated to distribute the cost to high‐risk areas that will use insurance more frequently than others. This approach aligns more with market‐based systems adopted in the United Kingdom or Germany rather than France, Switzerland, or Spain (Paudel 2012). Another area where adverse selection could play against the CFIP is the ceding mechanism between the Crown corporation and the insurance industry. Optional or partial transfer of an insurance company's obligations to the CFIP could incentivize the industry to retain the most profitable homeowners, exploiting any information asymmetry between the public reinsurer and the insurance market. A similar phenomenon occurs in the United States, where the National Flood Insurance Program competes with private industry in some areas (Elliott 2024). In aforementioned cases of adverse selection, continued investments in flood hazard and risk mapping in Canada can improve the price signal quality, reduce adverse selection, and help locate areas where risk reduction should be prioritized, in turn making flood insurance more viable in the long run.

We contribute to existing research on flood insurance in several ways. The article provides a first of its kind assessment of flood insurance design in Canada at a national scale that is relevant for comparison with other international assessments. The relatively high spatial concentration of risk in Canada demonstrates the challenge of managing compensation adequacy at the same time as affordability. Consistent with studies in Europe and the United States, the findings confirm that use of key design elements facilitated by a public–private arrangement between insurers and government can sustain affordability while offering adequate compensation (Bradt et al. 2021; Hudson et al. 2017, 2019). In addition, the article offers a novel methodology for evaluating how insurance design influences the objectives needed to demonstrate a public benefit. Most papers tend to focus on one objective such as risk reduction or affordability, whereas this study incorporated national flood model outputs to compare the relationship between several design elements and objectives. Replication of this methodology in other jurisdictions is dependent on data availability and assumptions in the underlying risk model but represents an important opportunity for comparison. For example, similar to the market exclusion proposed in a number of the scenarios, Germany's flood insurance system sharply limits coverage in high‐risk areas. In Canada, there is an opportunity to learn from and improve on this approach since the insurance model remains under development. Several provinces now offer “one and done” (or lifetime limit on) disaster assistance offers to people in high‐risk areas, which could be used to address the market exclusions. In the event of a flood, households can choose to use the assistance and rebuild but forgo future insurance and government funding, or they can choose to take a buyout leading to a permanent solution to reoccurring flood risk, further improving the affordability of coverage. Moreover, similar to the United Kingdom's Flood Re, Canada could consider a time limit on subsidization to encourage investment in risk mitigation and limit burdens on government budgets.

For policymakers, the article provides a useful architecture for decision‐making as the flood insurance program is being designed. Once implemented, there will be an opportunity to validate the use of national flood models to evaluate the public benefits of Canada's Flood Insurance Program in real‐time as premiums are set and claims are processed.

## Conflicts of Interest

The authors declare no conflicts of interest.

## Figures and Tables

**FIGURE A1.1:**
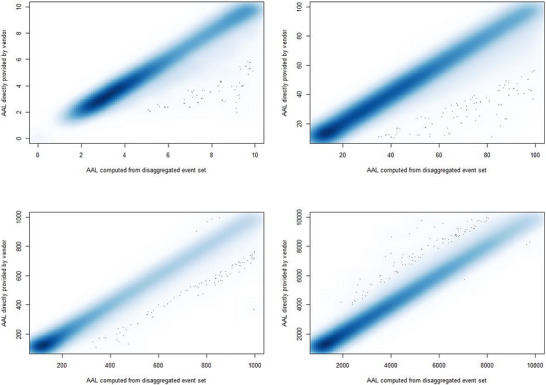
Correspondence between the AAL directly provided by KatRisk and the AAL computed from the disaggregated event set. AAL, average annual loss.

## Data Availability

The data that support the findings of this study are available from KatRisk, Verisk Canada, and Lightbox (DMTI Spatial). Restrictions apply to the availability of these data, which were used under license for this study.

## Endnotes

1 A dissemination area is a small, relatively stable geographic unit with an average population of 400–700 persons. It is the smallest standard geographic area for which all census data are disseminated (Statistics Canada).

2 A DB is an area bounded on all sides by roads and/or boundaries. They are the smallest areas for which population counts are reported in Canada (Statistics Canada).

3 The methodological choices made in this article are not designed for decision‐making at the building or neighborhood scale. Accurate micro‐scale flood risk assessment generally requires high‐resolution topographic, hydrological, and structural data that are beyond the scope of what we use here.

4 In unreported robustness tests, we assign the median after‐tax income to all households within each dissemination area. This alternative scenario has no material impact on the results in terms of trade‐offs between the competing policy objectives. A complete list of simulated flood‐insurance designs and their associated performance metrics under this alternative income attribution scheme is available from the authors upon request.

## Appendix A Disaggregation Algorithm

### A.1

Because of the high number of individual locations (over 9.8 million properties), the event set provided by KatRisk was aggregated over properties for each dissemination block (DB). However, to compute the effect of deductibles and limits for each homeowner, we need an individual loss distribution (per property). We therefore developed an approach to distribute simulated losses over each property of a DB.

An easy approach to spread flood losses over the properties of a DB would be to assume that each property suffers the same amount of loss. However, the exact location within the DB, along with the presence of a basement, which, in turn, affects the first‐floor elevation, are individual characteristics that influence the distribution of loss for each homeowner. Therefore, such a simple disaggregation approach would tend to overestimate (underestimate) the cost of insurance for low‐(high‐)risk properties within each DB.

Consequently, we have designed a disaggregation algorithm that distributes simulated losses proportional to the AAL. But given the heterogeneity of the AAL and of the reconstruction costs within a DB, a simple multiplication factor would often yield losses above the reconstruction costs, which is both unrealistic and undesired for large simulated events.

The algorithm is as follows (for each simulated yearly loss and each DB):

1. Compute the amount of simulated loss to distribute across homeowners of a given DB. At first, this is the simulated loss from the event set.
2. Compute the relative weight of each property based upon their AAL. At first, this is computed over all properties of the DB. Afterwards, this is computed on properties that remain.
3. To each property, then assign the minimum between the reconstruction cost of the home or the weight of the property times the amount of loss to distribute. If a loss has been assigned at previous rounds, we should deduce that amount from the reconstruction cost.
4. If 100% of the simulated loss has been assigned to properties, then stop. Otherwise, repeat the preceding steps, using the amount of simulated loss left to distribute.

The next example illustrates the process. Let us suppose the simulated loss is $1,750,000 for a fictitious DB with five properties. Table A1.1 provides the breakdown of calculations.

**TABLE A1.1:** Working example of the disaggregation algorithm.

Home #	Reconstruction costs ($)	AAL ($)	% AAL	Round #1		Round #2		Round #3		Loss (total, $)
				Weight (%)	Assigned loss ($)	Weight (%)	Assigned loss ($)	Weight (%)	Assigned loss ($)	
1	453,000	425	31.0	31.0	453,000	0.0	0	0.0	0	**453,000**
2	512,000	247	18.0	18.0	315,741	26.2	23,622	44.5	9997	**349,360**
3	512,000	389	28.4	28.4	497,261	41.2	14,739	0.0	0	**512,000**
4	453,000	126	9.2	9.2	161,066	13.3	12,050	22.7	5100	**178,216**
5	453,000	182	13.3	13.3	232,652	19.3	17,406	32.8	7366	**257,423**
				**100.0**	**1,659,720**	**100.0**	**67,817**	**100.0**	**22,463**	**1,750,000**

There are five dwellings in this DB whose reconstruction costs and AAL are given in Table A1.1. In round #1, the simulated loss to distribute in step 1 is, therefore, $1,750,000. The column Weight is the relative weight of each property's AAL. For example, property #1 accounts for 31% of the total AAL of the DB. The assigned loss in round #1 step 2 is the minimum between the reconstruction cost and the weight times $1,750,000. In the case of property #1, we cannot assign a loss larger than the reconstruction cost, and therefore, the assigned loss is $453,000. That means that not all of the $1,750,000 has been redistributed, and therefore, we need to continue with a second round to distribute $90,280 over the four remaining properties. We compute again the relative weight of each remaining property (excluding property #1 who suffered a loss of 100% of reconstruction cost). Then the assigned loss is the minimum between that weight times the remaining loss ($90,280) and any amount left, which is the reconstruction cost minus the assigned loss in round #1. After round #2, property #3 has suffered a loss of 100% of reconstruction cost. Finally, in round #3, there are $22,463 left to distribute among properties #2, 4, and 5. After three rounds, the original loss has been redistributed to all five properties.

The disaggregation algorithm has been validated by computing the AAL per homeowner using the disaggregated event set and compared with what was directly provided by KatRisk. Figure A1.1 shows the match between each homeowner's AAL and its equivalent computed from the disaggregated event set. We see that there is a clear correspondence between the two with a smooth scatter plot where darker tones of color show a greater concentration of points.

The same result holds in each panel that zooms in on various levels of AAL: between $0 and $10 in the top left, between $10 and $100 in the top right, between $100 and $1000 in the bottom left, and finally, between $1000 and $10,000 in the bottom right. The correlation between the points is 99.93%. Moreover, the Canada‐wide total AAL directly provided by KatRisk is $1.418 billion, whereas we get $1.413 billion with the disaggregation algorithm. The latter amount is within 99.62% of the original amount.

## Appendix B Complete List of Simulated Flood Insurance Designs With Associated Performance Criteria

**Table:**

					Affordability			Coverage		Participation	
					C1.1	C1.2	C1.3	C2.1	C2.2	C3	
	Subsidies	Take‐up	Deductible ($)	Limit ($)	Reasonable premium (%)	Expensive premium (%)	Mean PR‐INC ratio high risk (%)	Residual risk (%)	Mean uninsured loss high risk (%)	High‐risk Take‐up (%)	Cost of public assistance ($M)
1	None	Full Part.	0	None	95.8	3.2	5.4	0.0	0.0	100.0	0.0
2	None	Full Part.	1000	None	96.0	3.1	5.3	5.3	3.3	100.0	0.0
3	None	Full Part.	5000	None	96.2	2.9	5.1	15.4	12.5	100.0	0.0
4	None	Full Part.	10,000	None	96.5	2.8	4.8	23.2	21.1	100.0	0.0
5	None	Full Part.	25,000	None	96.9	2.4	4.2	37.7	38.8	100.0	0.0
6	None	Full Part.	1000	300,000	96.0	3.1	4.8	13.0	7.5	100.0	0.0
7	None	Full Part.	5000	300,000	96.3	2.9	4.6	23.1	16.7	100.0	0.0
8	None	Full Part.	10,000	300,000	96.5	2.7	4.3	30.9	25.3	100.0	0.0
9	None	Full Part.	25,000	300,000	97.0	2.4	3.7	45.4	43.0	100.0	0.0
10	Progressive	Full Part.	0	None	95.8	3.1	2.1	0.0	0.0	100.0	1091.7
11	Progressive	Full Part.	1000	None	96.0	3.0	2.1	5.3	3.3	100.0	1075.7
12	Progressive	Full Part.	5000	None	96.2	2.8	2.0	15.4	12.5	100.0	1022.1
13	Progressive	Full Part.	10,000	None	96.5	2.7	1.9	23.2	21.1	100.0	965.7
14	Progressive	Full Part.	25,000	None	96.9	2.4	1.7	37.7	38.8	100.0	829.7
15	Progressive	Full Part.	1000	300,000	96.0	3.0	2.0	13.0	7.5	100.0	903.1
16	Progressive	Full Part.	5000	300,000	96.3	2.8	1.9	23.1	16.7	100.0	850.0
17	Progressive	Full Part.	10,000	300,000	96.5	2.7	1.8	30.9	25.3	100.0	794.1
18	Progressive	Full Part.	25,000	300,000	97.0	2.3	1.6	45.4	43.0	100.0	659.4
19	Dollar cap	Full Part.	0	None	95.8	3.0	2.3	0.0	0.0	100.0	1059.9
20	Dollar cap	Full Part.	1000	None	96.0	2.9	2.3	5.3	3.3	100.0	1044.7
21	Dollar cap	Full Part.	5000	None	96.2	2.8	2.2	15.4	12.5	100.0	993.0
22	Dollar cap	Full Part.	10,000	None	96.5	2.6	2.1	23.2	21.1	100.0	937.6
23	Dollar cap	Full Part.	25,000	None	97.0	2.3	1.9	37.7	38.8	100.0	802.4
24	Dollar cap	Full Part.	1000	300,000	96.0	2.9	2.3	13.0	7.5	100.0	853.2
25	Dollar cap	Full Part.	5000	300,000	96.3	2.8	2.2	23.1	16.7	100.0	801.9
26	Dollar cap	Full Part.	10,000	300,000	96.5	2.6	2.1	30.9	25.3	100.0	747.1
27	Dollar cap	Full Part.	25,000	300,000	97.0	2.3	1.8	45.4	43.0	100.0	613.6
28	None	Random (80%)	0	None	95.8	3.2	5.4	20.0	20.0	80.0	0.0
29	None	Random (80%)	1000	None	96.0	3.1	5.3	24.2	22.6	80.0	0.0
30	None	Random (80%)	5000	None	96.2	2.9	5.1	32.4	30.0	80.0	0.0
31	None	Random (80%)	10,000	None	96.5	2.8	4.8	38.5	36.8	80.1	0.0
32	None	Random (80%)	25,000	None	96.9	2.4	4.2	50.1	51.0	80.1	0.0
33	None	Random (80%)	1000	300,000	96.0	3.1	4.8	30.4	26.0	79.9	0.0
34	None	Random (80%)	5000	300,000	96.3	2.9	4.6	38.6	33.4	80.0	0.0
35	None	Random (80%)	10,000	300,000	96.5	2.7	4.3	44.7	40.2	80.0	0.0
36	None	Random (80%)	25,000	300,000	97.0	2.4	3.7	56.3	54.4	80.1	0.0
37	Progressive	Random (80%)	0	None	95.8	3.1	2.1	19.9	20.0	80.0	874.8
38	Progressive	Random (80%)	1000	None	96.0	3.0	2.1	24.3	22.6	80.0	860.2
39	Progressive	Random (80%)	5000	None	96.2	2.8	2.0	32.3	30.0	80.0	817.9
40	Progressive	Random (80%)	10,000	None	96.5	2.7	1.9	38.5	36.9	80.0	773.4
41	Progressive	Random (80%)	25,000	None	96.9	2.4	1.7	50.2	51.0	80.0	663.4
42	Progressive	Random (80%)	1000	300,000	96.0	3.0	2.0	30.3	25.9	80.1	723.2
43	Progressive	Random (80%)	5000	300,000	96.3	2.8	1.9	38.4	33.3	80.1	681.5
44	Progressive	Random (80%)	10,000	300,000	96.5	2.7	1.8	44.6	40.2	80.1	637.2
45	Progressive	Random (80%)	25,000	300,000	97.0	2.3	1.6	56.4	54.5	80.0	526.0
46	Dollar cap	Random (80%)	0	None	95.9	3.0	2.3	20.0	20.0	80.0	848.5
47	Dollar cap	Random (80%)	1000	None	96.0	2.9	2.3	24.3	22.7	79.9	835.8
48	Dollar cap	Random (80%)	5000	None	96.2	2.8	2.2	32.4	30.0	80.0	793.6
49	Dollar cap	Random (80%)	10,000	None	96.5	2.6	2.1	38.5	36.8	80.1	751.1
50	Dollar cap	Random (80%)	25,000	None	97.0	2.3	1.9	50.2	51.1	80.0	641.3
51	Dollar cap	Random (80%)	1000	300,000	96.0	2.9	2.3	30.4	26.0	80.0	683.2
52	Dollar cap	Random (80%)	5000	300,000	96.3	2.8	2.2	38.5	33.3	80.0	642.1
53	Dollar cap	Random (80%)	10,000	300,000	96.5	2.6	2.1	44.7	40.2	80.0	598.6
54	Dollar cap	Random (80%)	25,000	300,000	97.0	2.3	1.8	56.4	54.5	80.0	490.4
55	None	Ex. very‐high risk	0	None	96.8	2.2	3.0	41.1	20.0	80.0	0.0
56	None	Ex. very‐high risk	1000	None	96.9	2.1	2.9	46.1	23.1	80.0	0.0
57	None	Ex. very‐high risk	5000	None	97.2	1.9	2.7	55.2	31.8	80.0	0.0
58	None	Ex. very‐high risk	10,000	None	97.5	1.8	2.5	61.9	39.8	80.0	0.0
59	None	Ex. very‐high risk	25,000	None	97.9	1.5	2.0	73.3	55.9	80.0	0.0
60	None	Ex. very‐high risk	1000	300,000	96.9	2.1	2.8	47.1	24.6	80.0	0.0
61	None	Ex. very‐high risk	5000	300,000	97.2	1.9	2.6	56.2	33.4	80.0	0.0
62	None	Ex. very‐high risk	10,000	300,000	97.5	1.8	2.4	62.8	41.3	80.0	0.0
63	None	Ex. very‐high risk	25,000	300,000	98.0	1.4	1.9	74.2	57.4	80.0	0.0
64	Progressive	Ex. very‐high risk	0	None	96.8	2.1	1.8	41.1	20.0	80.0	299.8
65	Progressive	Ex. very‐high risk	1000	None	96.9	2.0	1.7	46.1	23.1	80.0	290.1
66	Progressive	Ex. very‐high risk	5000	None	97.2	1.9	1.6	55.2	31.8	80.0	259.6
67	Progressive	Ex. very‐high risk	10,000	None	97.5	1.7	1.5	61.9	39.8	80.0	229.8
68	Progressive	Ex. very‐high risk	25,000	None	97.9	1.4	1.3	73.3	55.9	80.0	164.9
69	Progressive	Ex. very‐high risk	1000	300,000	96.9	2.0	1.7	47.1	24.6	80.0	272.6
70	Progressive	Ex. very‐high risk	5000	300,000	97.2	1.8	1.6	56.2	33.4	80.0	242.5
71	Progressive	Ex. very‐high risk	10,000	300,000	97.5	1.7	1.5	62.8	41.3	80.0	213.1
72	Progressive	Ex. very‐high risk	25,000	300,000	98.0	1.4	1.2	74.2	57.4	80.0	149.5
73	Dollar cap	Ex. very‐high risk	0	None	96.8	2.1	2.1	41.1	20.0	80.0	229.2
74	Dollar cap	Ex. very‐high risk	1000	None	96.9	2.0	2.1	46.1	23.1	80.0	221.1
75	Dollar cap	Ex. very‐high risk	5000	None	97.2	1.9	1.9	55.2	31.8	80.0	194.9
76	Dollar cap	Ex. very‐high risk	10,000	None	97.5	1.7	1.8	61.9	39.8	80.0	169.0
77	Dollar cap	Ex. very‐high risk	25,000	None	97.9	1.4	1.5	73.3	55.9	80.0	112.9
78	Dollar cap	Ex. very‐high risk	1000	300,000	97.0	2.0	2.1	47.1	24.6	80.0	201.9
79	Dollar cap	Ex. very‐high risk	5000	300,000	97.2	1.8	1.9	56.2	33.4	80.0	176.2
80	Dollar cap	Ex. very‐high risk	10,000	300,000	97.5	1.7	1.8	62.8	41.3	80.0	150.9
81	Dollar cap	Ex. very‐high risk	25,000	300,000	98.0	1.4	1.5	74.2	57.4	80.0	96.5
82	None	Ex. very expensive	0	None	98.2	0.8	1.4	61.3	48.1	51.9	0.0
83	None	Ex. very expensive	1000	None	98.3	0.7	1.4	65.7	50.0	52.6	0.0
84	None	Ex. very expensive	5000	None	98.5	0.7	1.3	72.9	55.2	54.8	0.0
85	None	Ex. very expensive	10,000	None	98.6	0.6	1.2	77.7	60.0	56.9	0.0
86	None	Ex. very expensive	25,000	None	98.8	0.5	1.0	84.9	69.6	61.8	0.0
87	None	Ex. very expensive	1000	300,000	98.3	0.8	1.4	65.5	50.1	53.4	0.0
88	None	Ex. very expensive	5000	300,000	98.4	0.7	1.3	72.7	55.3	55.6	0.0
89	None	Ex. very expensive	10,000	300,000	98.6	0.6	1.2	77.5	60.1	57.8	0.0
90	None	Ex. very expensive	25,000	300,000	98.8	0.6	1.0	84.7	69.8	62.8	0.0
91	Progressive	Ex. very expensive	0	None	96.6	2.3	1.8	34.2	16.2	83.8	411.0
92	Progressive	Ex. very expensive	1000	None	96.7	2.2	1.8	39.0	19.1	84.1	405.7
93	Progressive	Ex. very expensive	5000	None	97.0	2.1	1.7	47.1	27.1	84.9	391.4
94	Progressive	Ex. very expensive	10,000	None	97.2	2.0	1.6	53.0	34.5	85.8	375.2
95	Progressive	Ex. very expensive	25,000	None	97.5	1.8	1.4	62.4	49.3	87.9	340.8
96	Progressive	Ex. very expensive	1000	300,000	96.6	2.3	1.8	36.8	19.2	86.7	435.8
97	Progressive	Ex. very expensive	5000	300,000	96.9	2.2	1.7	45.1	27.3	87.6	420.0
98	Progressive	Ex. very expensive	10,000	300,000	97.1	2.1	1.6	50.9	34.7	88.5	402.9
99	Progressive	Ex. very expensive	25,000	300,000	97.4	1.9	1.4	60.4	49.7	91.0	367.9
100	Dollar cap	Ex. very expensive	0	None	97.3	1.6	1.7	32.4	29.1	71.0	542.4
101	Dollar cap	Ex. very expensive	1000	None	97.4	1.5	1.6	37.1	31.4	71.4	535.3
102	Dollar cap	Ex. very expensive	5000	None	97.6	1.4	1.5	45.6	38.0	72.8	511.3
103	Dollar cap	Ex. very expensive	10,000	None	97.8	1.4	1.4	51.7	44.2	74.1	485.6
104	Dollar cap	Ex. very expensive	25,000	None	98.1	1.2	1.2	62.1	56.9	77.1	423.0
105	Dollar cap	Ex. very expensive	1000	300,000	97.4	1.5	1.6	42.4	34.1	71.6	404.8
106	Dollar cap	Ex. very expensive	5000	300,000	97.6	1.4	1.5	50.8	40.7	73.0	381.1
107	Dollar cap	Ex. very expensive	10,000	300,000	97.8	1.3	1.4	56.9	46.9	74.3	355.9
108	Dollar cap	Ex. very expensive	25,000	300,000	98.1	1.2	1.2	67.3	59.6	77.4	294.4
